# High incidence and mortality of *Pneumocystis jirovecii* infection in anti-MDA5-antibody-positive dermatomyositis: experience from a single center

**DOI:** 10.1186/s13075-021-02606-8

**Published:** 2021-09-04

**Authors:** Linlin Huang, Qiong Fu, Yan Ye, Yanwei Lin, Qingran Yan, Sheng Chen

**Affiliations:** Department of Rheumatology, Renji Hospital, Shanghai Jiao Tong University School of Medicine, Shanghai, 200001 China

**Keywords:** Anti-MDA5-antibody-positive dermatomyositis, *Pneumocystis jirovecii* pneumonia, Incidence, Mortality, Treatment

## Abstract

**Background:**

Idiopathic inflammatory myopathies (IIM) are associated with a significantly higher risk of opportunistic infections including *Pneumocystis jirovecii* pneumonia (PJP), a potentially fatal opportunistic infection. However, no prior studies have evaluated PJP infection in subtypes of IIM.

**Objectives:**

To investigate the prevalence and mortality rate of PJP infection in subgroups of IIM patients stratified according to myopathy-specific antibodies.

**Methods:**

In the first part of the study, 463 consecutive patients with IIM were prospectively followed for a period of at least 1 year to analyze the incidence of PJP. In the second part of the study, we enrolled 30 consecutive PJP patients with any rheumatic disease in order to identify the mortality rate and risk factors by Cox regression analysis. The Kaplan-Meier method with log-rank testing was used to assess differences in survival.

**Results:**

The prevalence of PJP in IIM patients was found to be 3.0/100 person-years, while in MDA5^+^ DM patients it was 7.5/100 person-years and in MDA5^−^ IIM patients 0.7/100 person-years (*P* < 0.05). PJP typically occurred in the first 2 months in the case of MDA5^+^ DM patients who had a significant decrease in their CD4^+^ T cell counts and lymphocyte counts (*P* < 0.05). In PJP patients, 3-month mortality was higher for MDA5^+^ DM patients than in those with other rheumatic diseases (83.3% vs 38.9%, *P* < 0.05). Alarmingly, MDA5^+^ DM patients seemed not to benefit from prompt anti-PJP treatment, unlike patients with other rheumatic diseases whose survival improved when anti-PJP treatment was started within 6 days (*P* < 0.05).

**Conclusion:**

PJP has an alarming high incidence and mortality in MDA5^+^ DM patients. Timely treatment for PJP seems not to improve the prognosis of patients with this particular subtype. Hence, there remains a crucial unmet need to develop PJP prophylaxis for MDA5^+^ DM patients.

**Supplementary Information:**

The online version contains supplementary material available at 10.1186/s13075-021-02606-8.

## Key messages

1. The incidence of PJP in IIM patients is significantly higher in MDA5^+^ than MDA5^−^ subtype.
2. The PJP mortality of MDA5^+^ DM patients is higher than that in patients with other rheumatic diseases.
3. Timely anti-PJP treatment significantly improves the prognosis of PJP in rheumatic disease yet has no benefit for MDA5^+^ DM patients.
4. Our data suggest the necessity of developing PJP prophylaxis in MDA5^+^ DM patients, especially in the first 3 months of treatment or when the patient’s CD4^+^ T cell count decreases to < 200 cells/μL.

## Introduction

Patients with rheumatic disease receiving intensive immunosuppressive therapy, and who are therefore immunocompromised, often suffer from opportunistic infections [1]. The risk of opportunistic infection is highest for dermatomyositis/polymyositis (PM/DM) patients, followed by systemic lupus erythematosus, systemic sclerosis, rheumatoid arthritis, and finally primary Sjogren’s syndrome [2]. A recent study showed that underlying PM/DM significantly predisposes patients to *Pneumocystis jirovecii* pneumonia (PJP) [3]. This is a rare but potentially life-threatening opportunistic infection with a 30–60% mortality rate among immunocompromised (non-HIV) patients [4, 5]. In patients with rheumatic immune diseases, most PJP occurs in the first 3 months after initiating immunosuppressive therapy [2, 6].

The term idiopathic inflammatory myopathy (IIM) denotes a group of autoimmune diseases characterized by myasthenia and typical skin rash, among which PM and DM are the most common. Myositis-specific antibodies have long been identified and their value for stratifying patients with different outcomes has been recognized. However, there are very few reports on PJP in the different IIM subtypes. A recent study identified anti-melanoma differentiation-associated gene 5 antibody (anti-MDA5) as the only myositis-specific antibody that was associated with PJP in a multicenter juvenile DM cohort [7].

Unfortunately, MDA5^+^ DM is one of the subtypes of IIM with a poor prognosis and is mainly characterized by progressive interstitial lung disease, with or without muscle damage [8]. For adult MDA5^+^ patients, there appears to be only one report on two PJP+ deceased cases [9]. Hence, the mortality rate for PJP in MDA5^+^ patients is unknown. Therefore, in the present study, we first investigated the incidence of PJP in IIM patients with or without anti-MDA5 antibody and then analyzed the outcomes of anti-PJP treatment and mortality risk factors for PJP infection in different rheumatic diseases.

## Patients and methods

### Patients

In the first part of the study, we evaluated PJP incidence in an IIM cohort. All clinically diagnosed adult IIM patients fulfilling the 1997 classification criteria [10] were prospectively recruited from May 2017 to January 2020 at the Department of Rheumatology, Renji Hospital, China. Patients were screened for myositis-specific antibodies and myositis-associated antibodies using a commercial immunoblot assay with 16 autoantigens. Baseline characteristics of patients in the hospital, including demographic, clinical, and laboratory data, were acquired from the patients’ electronic medical records. Follow-up data were collected over a period of at least 1 year. The occurrence of PJP was evaluated in these patients.

In the second part of the study, anti-PJP treatment outcomes were evaluated in all PJP patients with rheumatic diseases. Thirty adult PJP patients with rheumatic diseases were recruited in a consecutive cohort study from May 2017 to January 2020 at the Department of Rheumatology, Renji Hospital, once the diagnosis of PJP was confirmed. Baseline characteristics including demographic, clinical, and laboratory data were acquired at the time of the patient’s first admission to the hospital. Follow-up data were collected over a period of at least 3 months. The 3-month cumulative survival rates were then evaluated. Written informed consent was obtained from each study participant. The study was conducted in accordance with the Declaration of Helsinki and was approved by the Ethics Committee of Renji Hospital, Shanghai, China (ID: 2013-126).

### Diagnosis of PJP

The diagnosis of PJP was based on comprehensive evaluation by clinical manifestations such as fever or acute dyspnea, characteristic radiographic findings, and etiological evidence. For confirmation, a case needed to have positive microbiological tests such as by next-generation sequencing and Grocott-Gomori methenamine-silver staining of bronchoalveolar lavage fluid. A probable case with typical manifestations but no etiological evidence needed confirmation by two infection specialists. However, a positive PJP sequencing result in the absence of clinical manifestations was not sufficient to define PJP infection.

### Statistical analysis

Statistical analysis was performed using the SPSS 23.0 software package (IBM Corp., Armonk, NY, USA) and GraphPad Prism 8.0 (GraphPad Software). We used the chi-square test or Fisher’s exact test to compare categorical variables and Student’s *t* test or the Mann-Whitney *U* test to compare continuous variables. Logistic regression was performed for multivariable analysis to identify independent risk factors for PJP occurrence and to calculate odds ratios. Cox proportional hazards regression was performed as a multivariable analysis to identify independent risk factors for death and to calculate hazard ratios. The optimal cut-off value was determined by using the receiver operating characteristic (ROC) analysis and the Kaplan-Meier method with log-rank testing was employed to assess differences in survival. For all analyses, two-tailed *P*-values less than 0.05 were considered statistically significant.

## Results

### PJP incidence in IIM patients

**Fig. 1:**
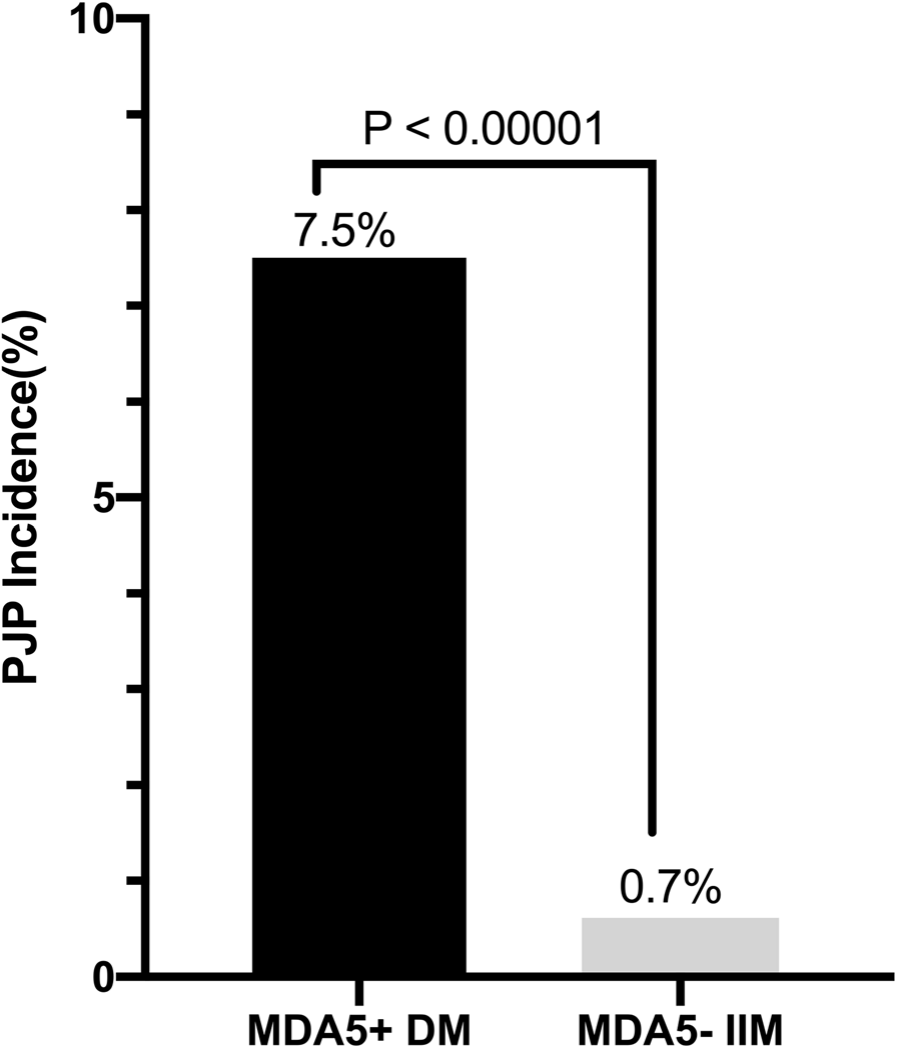
One-year PJP incidence in IIM patients. The incidence of MDA5^+^DM is 7.5/100 person-years, while MDA5^−^ IIM is 0.7/100 person-years. IIM, idiopathic inflammatory myopathy

There were 14 PJP patients in the IIM cohort (*n* = 463) after at least a 1-year follow-up (median follow-up 18 months, range 1–42 months). We calculated the prevalence of PJP in the IIM cohort at 3.0/100 person-years, while in MDA5^+^ DM patients, this rose to 7.5/100 person-years, in contrast to MDA5^−^ IIM patients where it was only 0.7/100 person-years (Fig. 1, 7.5% vs 0.7%, *P* < 0.00001). IIM patients were stratified into a PJP^+^ group and a PJP^−^ group to assess risk factors for PJP infection (Table 1). We found a significant imbalance in the distribution of anti-MDA5 positivity, which was higher in PJP^+^ IIM patients (85.7% vs 33.0%, *P* < 0.0001). In addition to clinical factors such as a shorter median course (2 months vs 6 months), interstitial lung disease (92.9% vs 70.4%), presence of diabetes (42.9% vs 13.6%), and higher prednisone exposure (50 mg vs 30 mg), we also found that laboratory parameters such as higher erythrocyte sedimentation rate and ferritin value, lower serum albumin, CD4^+^ T lymphocyte count, and overall lymphocyte count were all significantly different between PJP^−^ and PJP^+^ IIM patients (*P* < 0.05).

**Table 1:** Comparison of risk factors in PJP and non-PJP cases for IIM patients

	PJP (*n* = 14)	Non-PJP (*n* = 449)	*P* value
MDA5^+^ DM, *n* (%)	12 (85.7%)	148 (33.0%)	0.000
Non-MDA5 IIM, *n* (%)	2 (14.3%)	301 (67.0%)	
Male gender, *n* (%)	6 (42.9%)	135 (30.1%)	0.376
Assessed age, mean ± SD	54 ± 10	53 ± 12	0.635
Disease duration, years, median	2	6	0.001
ILD (%)	13 (92.9%)	316 (70.4%)	0.077
Premedication (last 1 month), *n* (%)			
Corticosteroid (≥20 mg pred, ≥ 1 month)	10 (71.4%)	226 (50.4%)	0.12
Corticosteroid, mg, median	50	30	0.001
Cyclophosphamide	2 (14.3%)	33 (7.3%)	0.286
Methotrexate	0 (0%)	37 (8.3%)	0.616
Azathioprine	0 (0%)	32 (7.1%)	0.613
Cyclosporine	3 (21.4%)	69 (15.4%)	0.465
Tacrolimus	4 (28.6%)	50 (11.1%)	0.068
Mycophenolate mofetil	0 (0%)	24 (5.3%)	1.000
Hydroxychloroquine	4 (28.6%)	95 (21.2%)	0.511
Biologics	1 (7.1%)	16 (3.6%)	0.412
Others	1 (7.1%)	76 (16.9%)	0.482
Diabetes, *n* (%)	6 (42.9%)	61 (13.6%)	0.009
ESR, mm/h, median	34.5	21	0.085
Creatine kinase, U/L, median	26.5	67.5	0.02
LDH, U/L, median	464.5	321	0.028
Ferritin, μg/mL, median	1122	341	0.001
Pre-albumin, g/L, median	208.5	214	0.832
Albumin, mg/L, median	29.4	32.5	0.005
CD4^+^ T cell counts at admission ×10^9^/L, median	113.4	350.4	0.000
Lymphocyte counts at admission ×10^9^/L, median	0.695	0.9	0.004

*ILD* interstitial lung disease, *ESR* erythrocyte sedimentation rate, *LDH* lactic dehydrogenase, *CK* creatine kinase

Considering the poor prognosis of MDA5^+^ DM, the requirement for aggressive treatment may blur the identification of risk factors for PJP infection. As shown in Table S1 (online), MDA5^+^ DM patients in this cohort did receive higher doses of corticosteroids and much more immunosuppression (IS) using agents such as cyclosporine, tacrolimus, and biologics. To identify risk factors, we used a logistic regression model and included factors with *P* values < 0.15 in dichotomous comparisons between PJP^+^ and PJP^−^ IIM patients, such as corticosteroid dose, tacrolimus use, CD4^+^ T cell counts, albumin level, presence of diabetes, MDA5^+^ DM, disease duration, and interstitial lung disease. As shown in Table 2, only the presence of anti-MDA5 antibody and low CD4^+^ T cell counts were identified as independent risk factors for PJP occurrence by this multivariate analysis.

**Table 2:** Risk factors for PJP occurrence in IIM patients

Variable	Univariable		Multivariable	
	OR (95% CI)	***P*** value	OR (95% CI)	***P*** value
Corticosteroid dose, mg	1.005 (0.999, 1.012)	0.117	-	0.703
Corticosteroid (≥20 mg, ≥ 1 month)	1.76 (0.581, 5.335)	0.317	*	*
Diabetes	4.758 (1.596, 14.186)	0.005	-	0.067
Tacrolimus	2.865 (0.869, 9.451)	0.084	-	0.616
Mycophenolate mofetil	1.157 (0.146, 9.162)	0.890	*	*
Ciclosporin	2.027 (0.619, 6.637)	0.243	*	*
Cyclophosphamide	0.809 (0.103, 6.347)	0.840	*	*
Methotrexate	0.000 (0.000, 0.000)	0.998	*	*
Biological agent	0.000 (0.000, 0.000)	0.999	*	*
MDA5^+^ DM	12.203 (2.696, 55.229)	0.001	6.374 (1.368, 29.722)	0.018
Age	1.006 (0.963, 1.050)	0.790	*	*
Interstitial lung disease	5.472 (0.709, 42.249)	0.103	-	0.742
Erythrocyte sedimentation rate	1.001 (0.997, 1.005)	0.659	*	*
Lactic dehydrogenase	1.000 (1.000, 1.001)	0.269	*	*
CD4 + T cell counts at admission	0.991 (0.985, 0.996)	0.001	0.986 (0.992, 0.997)	0.003
Lymphocyte counts at admission	0.099 (0.019, 0.522)	0.006	-	0.589
Disease duration, months, median	0.763 (0.600, 0.970)	0.027	-	0.066
Albumin	0.844 (0.761, 0.937)	0.001	-	0.667

CI confidence interval

*Not included in the multivariable model due to the lack of significant association in the univariable analysis

- Included in the multivariable model but lacking of significant association in the multivariate analysis

In an additional comparison between PJP^−^ and PJP^+^ patients with similar treatment and diabetes status among the MDA5^+^ DM patients (Table S2), PJP occurred at a median of 2 months and at the time of a clear decrease of CD4^+^ T cell count and lymphocyte count.

### PJP infection in cohorts with rheumatic diseases

To evaluate the impact of anti-MDA5 positivity on mortality caused by PJP, we further analyzed all PJP^+^ patients (*n* = 30) admitted to our medical center during the same period as the IIM cohort. Clinical features compared between MDA5^+^ DM patients and those with other rheumatic diseases are shown in Supplementary Table S3 (online), with more details of the 30 PJP^+^ patients provided in Supplementary Table S4 (online).

**Fig. 2:**
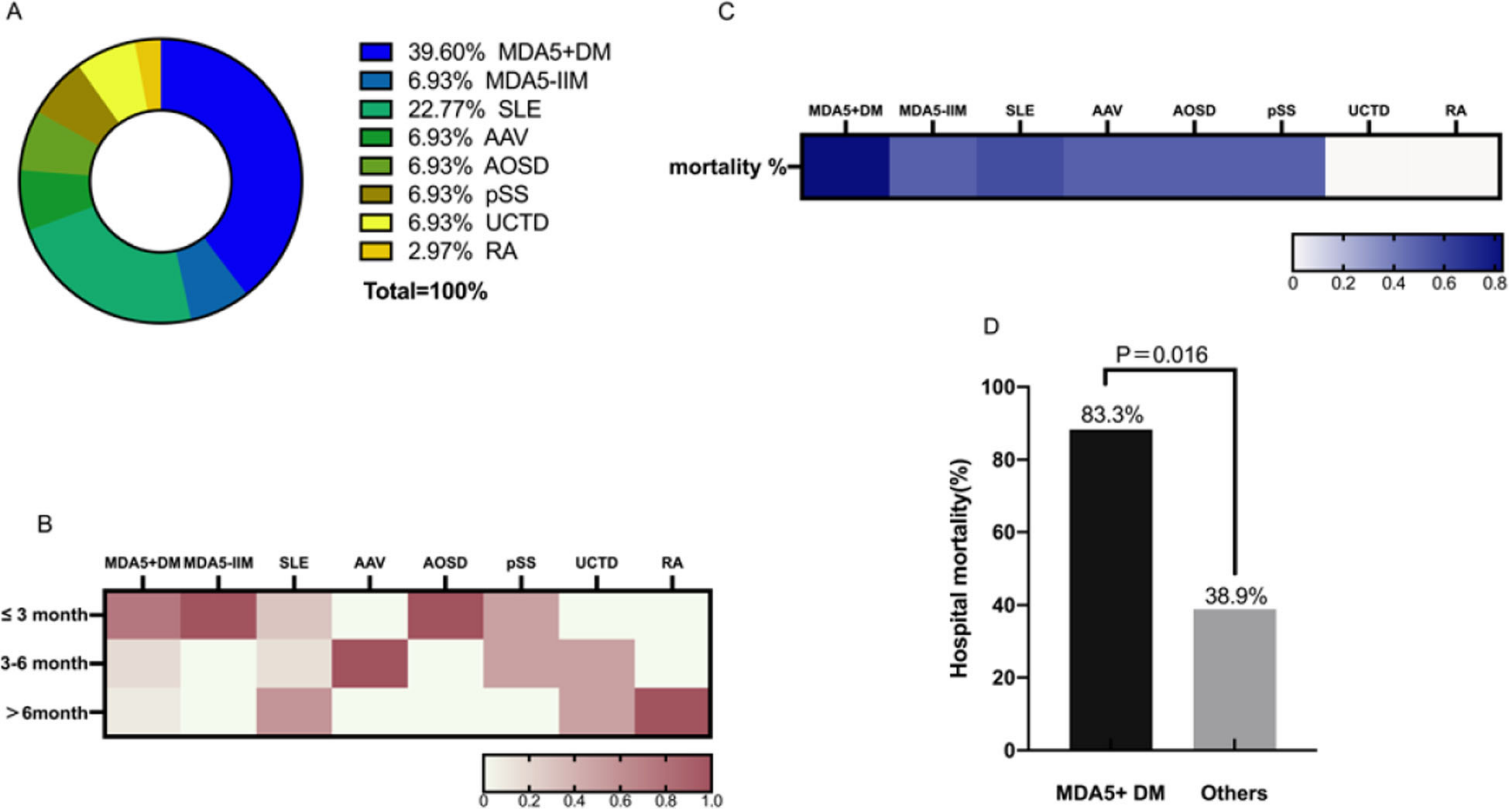
The clinical characters and mortality of the PJP cohort. This cohort contains all PJP cases from our center in the recent 3 years. **A** The distribution of underlying rheumatic diseases in all 30 PJP+ patients in this cohort. **B** The distribution of rheumatic disease duration at PJP onset. **C** The mortality of PJP cases in each rheumatic disease. **D** Comparison of PJP-associated mortality in MDA5^+^ DM and other rheumatic diseases

As shown in Fig. 2A, MDA5^+^ DM constituted the greater part of the PJP^+^ patients (*n* = 12, 39.60%), followed by systemic lupus erythematosus (7, 22.70%), MDA5^−^ IIM (2, 6.93%), ANCA-associated vasculitis (AAV; 2, 6.93%), adult-onset Still’s disease (AOSD; 2, 6.93%), primary Sjogren’s syndrome (pSS; 2, 6.93%), undifferentiated connective tissue disease (UCTD; 2, 6.93%), and finally, a single patient with rheumatoid arthritis (RA; 2.97%). The heatmap in Fig. 2B shows the duration of rheumatic disease at the time that PJP infection occurred. For IIM, AAV, and AOSD patients, PJP infection mostly occurred within 6 months of disease onset, while a little unexpectedly for SLE patients, PJP seemed to occur at any stage of disease in this cohort.

### PJP mortality

The mortality of anti-MDA5 antibody-positive patients was higher than that in other rheumatic diseases (83.3% vs 38.9% *P* = 0.016), as shown in Fig. 2C and D. We identified age, CD4^+^ T cell counts, and MDA5^+^ DM as risk factors for 3-month mortality at *P* < 0.10 by univariate analysis. These 3 factors were then analyzed in the Cox regression model, which confirmed that MDA5^+^ DM and CD4^+^ T cell counts were independent risk factors for mortality. Notably, of these two variables, despite having been previously identified as PJP mortality factors [11], CD4^+^ T cell counts only yielded a hazard ratio of 0.994 (95% CI 0.989–1.000) while MDA5^+^ DM had a much higher HR of 3.254 (95% CI 1.209–8.756) (Table 3).

**Table 3:** Risk factors for 3-month mortality of PJP with rheumatic disease

	Univariable		Multivariable	
Clinical factors	HR (95% CI)	*P* value	HR (95% CI)	*P* value
Assessed age	0.981 (0.951–1.012)	0.225	0.975 (0.940–1.010)	0.158
Gender	0.437 (0.207–0.923)	0.798	*	*
MDA5^+^ DM	2.830 (1.067–7.505)	0.037	3.254 (1.209–8.756)	0.02
AAV	0.674 (0.089, 5.099)	0.702	*	*
SLE	1.062 (0.346, 3.265)	0.916	*	*
CD4^+^ T cell counts	0.995 (0.989–1.001)	0.083	0.994 (0.989–1.000)	0.04
Lymphocyte counts	0.142 (0.025–0.808)	0.028	*	*
Interstitial lung disease	1.537 (0.540–4.375)	0.421	*	*
Steroid dose	0.291 (0.063, 1.350)	0.115	*	*
Cyclophosphamide	1.807 (0.586, 5.568)	0.303	*	*
Rituximab	0.854 (0.113, 6.450)	0.878	*	*
Time to anti-PJP treatment	1.577 (0.607–4.099)	0.35	*	*
Disease duration	1.003 (0.988–1.018)	0.677	*	*

*CI* confidence interval, *AAV* ANCA-associated vasculitis, *SLE* systemic lupus erythematosus

*Not included in the multivariable model due to the lack of significant association in the univariable analysis

**Fig. 3:**
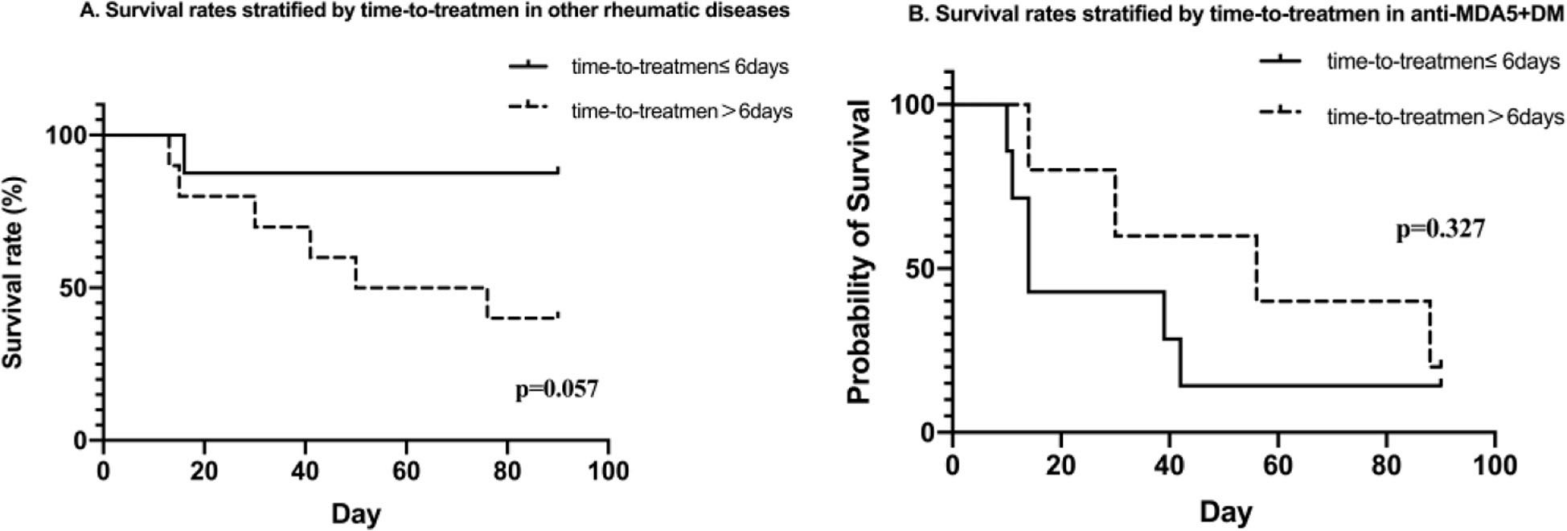
Survival stratified by time-to-anti-PJP-treatment in PJP patients. **A** Survival stratified by time-to-treatment in PJP with rheumatic disease other than MDA5^+^ DM. **B** Survival stratified by time-to-treatment in PJP with MDA5^+^ DM. Unlike patients with other rheumatic diseases, MDA5^+^ DM patients seemed not to benefit from prompt anti-PJP treatment

In addition to baseline risk factors, prompt anti-PJP treatment is a critical influence on patient survival. It has long been recognized that early diagnosis and treatment can improve the prognosis of PJP patients [12, 13]. We sought the relevant time limitation for optimal PJP treatment using ROC analysis, but the best cut-off value was not statistically significant (Supplementary Figure S1A, *P* = 0.0983, at a cut-off value of 7 days). When we further stratified all patients by anti-MDA5 status, a similar ROC as seen in previous reports was obtained for patients with rheumatic diseases other than MDA5^+^ DM. The time-to-PJP-treatment cut-off of 6 days exhibited an 85.7% sensitivity and 63.6% specificity, with an area under the curve (AUC) of 81.2% by ROC analysis. Thus, the time of 6 days was the optimal cut-off point for timely treatment of PJP (Supplementary Figure S1B, *P* = 0.0297). However, in MDA5^+^ DM patients, ROC analysis was uninformative (Figure S1C). We then analyzed patient survival to confirm the cut-off value. Consistent with previous reports, patients with other rheumatic diseases tended to have better survival if they received anti-PJP treatment within 6 days of the appearance of the first symptoms (Fig. 3A, 1-year survival rate 87.5% vs 40%, *P* = 0.057). However, in MDA5^+^ DM patients, there was no impact on survival no matter when treatment was started (Fig. 3B, *P* = 0.327). Thus, timely anti-PJP treatment did not benefit MDA5^+^ DM patients and increase their survival in our center.

## Discussion

Early in 1996, a previous study indicated that the prevalence of PJP in systemic lupus erythematosus patients was 1.7% while in DM patients it was 37.5%, but this was based on a very small cohort (*n* = 75) [14]. Our study is the first to report the incidence of PJP in adult MDA5^+^ DM patients in an independent cohort and is the largest study so far. For the first time, we describe the different incidences of PJP in anti-MDA5-positive and anti-MDA5-negative IIM patients. The incidence rate in MDA5^+^ DM patients is as much as 7.5/100 person-years in our cohort. A Cochrane review recommends that prophylactic treatment should be given when the risk of PJP infection in non-HIV immunocompromised individuals is greater than 6.2/100 person-years [15].

The reason why MDA5^+^ DM patients are more susceptible to infection by *Pneumocystis jirovecii* is still unknown. According to previous reports, high risk factors for rheumatic disease complicated with PJP included granulomatosis with polyangiitis; microscopic polyangiitis; autoimmune interstitial pneumonia; use of high-dose glucocorticoids, cyclophosphamide [16], or high-dose methotrexate; being older; having diabetes or nutritional deficiency; and severe lymphocytopenia or low CD4^+^ T cell counts [17, 18]. Similar to the reports above, comparisons in the present study showed that PJP^+^ IIM patients had been receiving higher doses of corticosteroids and had a higher prevalence of diabetes, lower albumin level, and low CD4^+^ T cell counts. However, in the multivariate analysis, only the MDA5^+^ DM disease subtype and low CD4^+^ T cell counts were identified as independent risk factors for PJP occurrence. In a comparison of PJP^−^ and PJP^+^ MDA5^+^ DM patients with similar characteristics and medication, a shorter disease duration prior to infection was noticed, as well as low CD4^+^ T cell counts in infected patients. These findings indicate that we need to pay more attention to PJP in MDA5^+^ DM patients, especially in the first 3 months after disease onset, or in patients with CD4^+^ T cell counts of < 200 cells/μL.

For predicting prognosis, baseline lung involvement has been suggested as a possible factor leading to fatal interstitial pneumonia in PJP^+^ RA patients [19]. Not surprisingly, CD4^+^ T cell cytopenia predicts mortality in kidney transplant patients with PJP, according to a recent report by Freiwald et al. [20]. Our study reports for the first time that the 3-month mortality rate of adult MDA5^+^ DM patients was as high as 83.3%. MDA5^+^ DM and CD4^+^ T cell counts were identified by multivariate analysis in our study as independent risk factors for mortality. Given the extremely high prevalence and poor outcome of ILD in MDA5^+^ DM patients, we speculate that lung comorbidity may contribute to lethality in PJP^+^ MDA5^+^ DM patients.

One important clinical outcome that was first noticed in our study is that timely PJP treatment did not improve the prognosis of MDA5^+^ DM patients, which is not the case in other non-HIV PJP infections. Although the number of PJP cases is low in our study, it is alarming enough to encourage us to take measures to deal with such a dangerous condition, in which prophylaxis against PJP may be a good approach [21]. It has been reported that prophylaxis against PJP in rheumatic patients can significantly reduce the incidence of the infection without severe adverse events [22]. The prophylaxis rate was only 4% (*n* = 19) of patients at admission in our IIM cohort, and merely 43 (9.3%) patients had been given any continuous anti-PJP prophylaxis since starting treatment for IIM in our hospital. There are currently no recommendation guidelines for PJP management in MDA5^+^ DM patients. The data presented here suggest the necessity of a further study of PJP prophylaxis in MDA5^+^ DM patients who may need this on a routine basis.

Limitations of this study include the following. First, the evaluation of PJP occurrence in IIM was observational, a limitation inherent to such studies. Second, the number of PJP cases was rather small, making any inferences from the regression model for independent PJP occurrence factors in IIM or MDA5^+^ DM potentially less robust. Third, the number of patients on PJP prophylactic treatment was too small for us to assess the benefit of trimethoprim-sulfamethoxazole treatment in MDA5^+^ DM patients. Fourth, patients in the IIM cohort were followed up for longer than those with other rheumatic diseases; this may introduce a bias when performing the mortality analysis. Therefore, a randomized controlled trial of PJP prophylaxis is needed.

## Conclusions

Here, we showed that MDA5^+^ DM patients are highly susceptible to infection with *Pneumocystis jirovecii*, which is also harder to cure than in other rheumatic diseases. The reason for the higher incidence and mortality may be related to the lower CD4^+^ T cell counts and progressive interstitial lung disease in MDA5^+^ patients. These findings suggest the necessity for a further systematic study of PJP prophylaxis in MDA5^+^ DM patients.

## Supplementary Information

**Additional file 1: Supplementary figure S1.** ROC curve for time-to-treatment cut-off valve. A. ROC curve for time-to-treatment cut-off valve in all PJP patients(P = 0.0983). B. ROC curve for time-to-treatment cut-off valve in PJP patients without MDA5^+^DM. Time-to-PJP-treatment cut-off point of 6 days showed 85.7% sensitivity and 63.6% specificity and with the Area Under Curve (AUC) 81.2%. The time of six-day was the optimal cut-off point for timely treatment for PJP (P = 0.03). C. ROC curve for time-to-treatment cut-off valve in all PJP patients (P > 0.999).

**Additional file 2: Supplementary table S1.** Patients characteristics and PJP infection rate in MDA5^+^DM and MDA5^-^ IIM patients. To show more detail of PJP patient in our cohort.

**Additional file 3: Supplementary table S2.** Comparison of risk factors in anti-MDA5-ab-positive patients. PJP occurred in a median time of 2 months and with obvious decrease of CD4^+^ T cell counts and lymphocytes.

**Additional file 4: Supplementary table S3.** Characteristics of patients with rheumatic disease and PJP infection from a single center. Compared to other rheumatic disease, PJP with MDA5^+^DM were characterized as higher percentage of ILD and PJP occurred earlier during the disease duration.

**Additional file 5: Supplementary table S4.** Clinical features of the 30 PJP cases at diagnosis. To show more detail of PJP patient in our cohort.

## Data Availability

All data generated or analyzed during this study are included in this published article [and its supplementary information files].

## Abbreviations

MDA5^+^ DM: Anti-melanoma differentiation-associated gene 5 antibody-positive dermatomyositis
MDA5^−^ IIM: Anti-melanoma differentiation-associated gene 5 antibody-negative idiopathic inflammatory myopathy
PJP: *Pneumocystis jirovecii* pneumonia
IIM: Idiopathic inflammatory myopathy
PM: Polymyositis
TMP-SMX: Trimethoprim/sulfamethoxazole
IS: Immunosuppressant
