# Supplementary figures and images for: High incidence and mortality of Pneumocystis jirovecii infection in anti-MDA5-antibody-positive dermatomyositis: experience from a single center
Source: Arthritis Res Ther. 2021 Sep 4;23:232. doi: 10.1186/s13075-021-02606-8 (PMC8417987; doi:10.1186/s13075-021-02606-8)

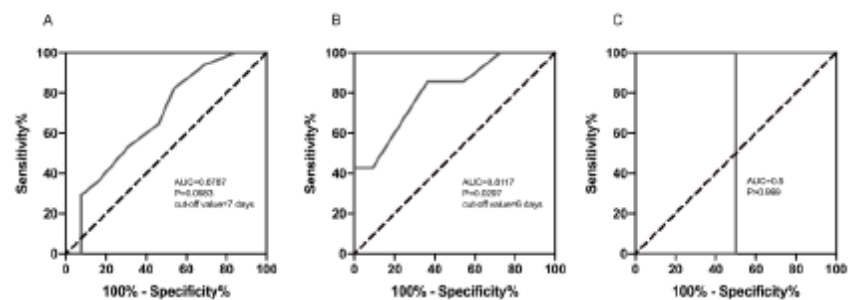

Supplementary figureS1.ROC curve for time-to-treatment cut-off valve

Supplement: Supplementary file 1 — Additional file 1: Supplementary figure S1. ROC curve for time-to-treatment cut-off valve. A. ROC curve for time-to-treatment cut-off valve in all PJP patients(P = 0.0983). B. ROC curve for time-to-treatment cut-off valve in PJP patients without MDA5+DM. Time-to-PJP-treatment cut-off point of 6 days showed 85.7% sensitivity and 63.6% specificity and with the Area Under Curve (AUC) 81.2%. The time of six-day was the optimal cut-off point for timely treatment for PJP (P = 0.03). C. ROC curve for time-to-treatment cut-off valve in all PJP patients (P > 0.999). [file 13075_2021_2606_MOESM1_ESM.pdf]
