# Supplementary material for: High incidence and mortality of Pneumocystis jirovecii infection in anti-MDA5-antibody-positive dermatomyositis: experience from a single center
Source: Arthritis Res Ther. 2021 Sep 4;23:232. doi: 10.1186/s13075-021-02606-8 (PMC8417987; doi:10.1186/s13075-021-02606-8)
Supplement: Supplementary file 2 — Additional file 2: Supplementary table S1. Patients characteristics and PJP infection rate in MDA5+DM and MDA5- IIM patients. To show more detail of PJP patient in our cohort. [file 13075_2021_2606_MOESM2_ESM.pdf]

Table S1 Patient characteristics and PJP infection rate in MDA5$^+$DM and MDA5- IIM patients

	MDA5$^+$DM (n=160)	MDA5$^-$IIM (n=303)	P value
male gender， n(%)	49(30.6%)	91(30.4%)	0.954
onset ages, mean ± SD	52±10	51±14	0.372
assess age, mean ± SD	53±10	53±13	0.689
Disease duration, month, median	4	9.5	0.000
ILD(%)	150(93.8%)	179(59.1%)	0.000
Premedication (last one month)， n (%)			
Corticosteroid(n, %) (≥20mg pred， ≥1month)	97(60.6%)	139(45.9%)	0.003
Cyclophosphamide	16(10.0%))	19(6.3%)	0.195
Methotrexate	3(1.9%)	34(11.3%)	0.000
Azathioprine	5(3.1%)	27(8.9%)	0.020
Cyclosporine	32(20.0%)	40(13.2%)	0.060
Tacrolimus	30(18.8%)	24(7.9%)	0.001
Mycophenolate mofetil	13(8.1%)	11(3.6%)	0.047
hydroxychloroquine	31(19.4%)	68(22.4%)	0.476
Biologics	10(6.3%)	7(2.31%)	0.032
others	31(19.4%)	46(15.2%)	0.294
Diabetes	35(22%)	32(10.6%)	0.001
ESR, mm/h, median	29	17	0.000
CK, U/L, median	30.0	163.5	0.000
LDH, U/L, median	309.0	338.5	0.440
Ferritin, ug/ml， median	818.7	230.6	0.000
Pre-albumin, g/L,median	208	215	0.482
Albumin, mg/L,median	31.3	33.6	0.001
CD4$^+$ T cell counts at admission cells/uL,median	249.6	427.8	0.000
Lymphocyte counts at admission x10^9/L,median	0.72	1.02	0.000
PJP infection (n, %)	12(7.5%)	2(0.7%)	0.000

ILD: interstia lung disease ESR:erythrocyte sedimentation rate
LDH:lactic dehydrogenase CK: Creatine kinase
