# Supplementary material for: High incidence and mortality of Pneumocystis jirovecii infection in anti-MDA5-antibody-positive dermatomyositis: experience from a single center
Source: Arthritis Res Ther. 2021 Sep 4;23:232. doi: 10.1186/s13075-021-02606-8 (PMC8417987; doi:10.1186/s13075-021-02606-8)
Supplement: Supplementary file 3 — Additional file 3: Supplementary table S2. Comparison of risk factors in anti-MDA5-ab-positive patients. PJP occurred in a median time of 2 months and with obvious decrease of CD4+ T cell counts and lymphocytes. [file 13075_2021_2606_MOESM3_ESM.pdf]

Supplementary table S2 Comparison of PJP+ between PJP- in MDA5^{+}DM patients

	PJP (n=12)	Non-PJP (n=148)	P value
male gender, n(%)	5(41.7%)	44(29.7%)	0.388
onset ages, mean ± SD	52±10	52±13	0.830
assess age, mean ± SD	53±10	53±12	0.988
Disease duration, month, median	2	4.5	0.022
ILD,n(%)	12(100%)	138(93.2%)	1.000
Predmedication (last one month), n (%)			
Corticosteroid. (≥20mg pred ≥1month)	9(75.0%)	88(59.5%)	0.368
Cyclophosphamide	2(16.7%)	14 (9.5%)	0.342
Methotrexate	0(0%)	3(2.0%)	1.000
Azathioprine	0(0%)	5(3.4%)	1.000
Cyclosporine	3(25%)	29(19.6%)	0.708
Tacrolimus	4(33.3%)	26(17.6%)	0.240
Mycophenolate Mofetil	0(0%)	13(8.8%)	0.601
hydroxychloroquine	2(16.7%)	29(19.6%)	1.000
Biologics	1(8.3%)	9(6.1%)	0.552
Others	1(8.3%)	30(20.3%)	0.464
Diabetes, n (%)	4(33.3%)	31(21.1%)	0.300
ESR, mm/h, median	34	29	0.588
CK, U/L, median	26	30	0.845
LDH, U/L, median	464.5	304.5	0.008
Ferritin, ug/ml,median	1120	808	0.138
Pre-albumin, g/L,median	220.5	207	0.271
Albumin, mg/L,median	29.9	31.4	0.153
CD4^{+} T cell counts at admission cells/uL,median	113.4	253.85	0.007
Lymphocyte counts at admission x10^9/L,median	0.695	0.720	0.127

ILD: interstia lung disease   ESR:erythrocyte sedimentation rate

LDH:lactic dehydrogenase   CK: Creatine kinase
