# Supplementary material for: High incidence and mortality of Pneumocystis jirovecii infection in anti-MDA5-antibody-positive dermatomyositis: experience from a single center
Source: Arthritis Res Ther. 2021 Sep 4;23:232. doi: 10.1186/s13075-021-02606-8 (PMC8417987; doi:10.1186/s13075-021-02606-8)
Supplement: Supplementary file 4 — Additional file 4: Supplementary table S3. Characteristics of patients with rheumatic disease and PJP infection from a single center. Compared to other rheumatic disease, PJP with MDA5+DM were characterized as higher percentage of ILD and PJP occurred earlier during the disease duration. [file 13075_2021_2606_MOESM4_ESM.pdf]

Supplementary table S3 **Characteristics of patients with rheumatic disease and PJP infection**

	MDA5+DM (n=12)	others* (n=18)	P value
male gender， n(%)	6(50%)	3(16.7%)	0.102
onset ages, mean ± SD	52±11	52±20	0.977
assess age, mean ± SD	52±11	54±18	0.715
Disease duration, month, median	2.25	4.5	0.018
PJP infection to induction therapy，month,median	1.25	2	0.18
cough,n(%)	10(83.3%)	12(66.7%)	0.419
shortness of breath,n(%)	12(100%)	16(88.9%)	0.503
ILD,n(%)	11(91.7%)	8(44.4%)	0.018
(1→3)-β-D-glucan, pg/Ml,median	125.25	147.30	0.363
LDH,U/L,median	361	521	0.356
$CD4^+$Tcell count,cells/uL,median	139	111	0.672
lymphocyte count, x10^9/L,median	0.5	0.5	0.433
HRCT			
diffuse ground-glass opacity,n(%)	10(83.3%)	13(72.2%)	0.699
diffuse infiltration,n(%)	10(83.3%)	15(83.3%)	1.000
etiology evidence,n(%)	10(83.3%)	10(55.6%)	0.235
Medications, n (%)			
Corticosteroid	10(83.3%)	13(72.2%)	0.699
hydroxychloroquine	10(83.3%)	15(83.3%)	0.210
No immunosuppressant	5(41.7%)	3(16.7%)	0.235
1 immunosuppressant	5(41.7%)	10(55.6%)	0.456
2 immunosuppressant	1(8.3%)	4(22.6%)	0.317
≥3immunosuppressant	1(8.3%)	1(5.6%)	1.000
biological agents	0(0%)	2(11.1%)	0.503
Mortality,n (%)	10(83.3%)	7(38.9%)	0.016

ILD: interstia lung disease

LDH:lactic dehydrogenase
