# Supplementary material for: High incidence and mortality of Pneumocystis jirovecii infection in anti-MDA5-antibody-positive dermatomyositis: experience from a single center
Source: Arthritis Res Ther. 2021 Sep 4;23:232. doi: 10.1186/s13075-021-02606-8 (PMC8417987; doi:10.1186/s13075-021-02606-8)
Supplement: Supplementary file 5 [file 13075_2021_2606_MOESM5_ESM.docx]

Table S4. Clinical features of the 30 PJP cases at diagnosis

Case	Gender	Age	Duration	Underlying disease#	Steroid dose mg	immunosuppressants	$CD4^+$ T cell counts cells/uL	Lymphocyte count *10^9/L	Hospital death
1	Male	65	2	$MDA5^+$DM	0	HCQ,CsA,Thd	189.54	0.26	YES
2	Female	60	2	**anti-TIFTY-ab+PM**	50	HCQ	18.72	0.12	YES
3	Female	36	4	$MDA5^+$DM	50	None	261.80	0.85	YES
4	Male	54	2	$MDA5^+$DM	50	Tac	185.60	0.58	YES
5	Female	69	2	$MDA5^+$DM	0	HCQ	33.84	0.12	YES
6	Female	50	1	$MDA5^+$DM	100	None	463.31	1.07	NO
7	Female	29	84	SLE	50	HCQ,MMF	105.27	1.21	NO
8	Male	46	3	$MDA5^+$DM	60	CsA	113.80	0.64	YES
9*	Female	38	3	AOSD	30	HCQ,RTX,Tac	157.59	0.51	YES
10	Female	54	9	$MDA5^+$DM	50	Tac	51.90	0.15	YES
11	Female	68	5	AAV	300	HCQ,Thd	96.30	0.30	YES
12*	Male	87	2	SLE	50	HCQ	96.75	2.25	NO
13	Female	71	4	AAV	50	CTX,RTX	249.00	0.50	NO
14	Male	56	6	pSS	20	HCQ,MTX	198.72	0.96	NO
15*	Female	73	3	AOSD	50	HCQ,CsA,Thd	117.04	0.56	NO
16	Female	29	4	SLE	100	HCQ,MMF	121.27	0.67	NO
17	Male	43	2.5	$MDA5^+$DM	50	None	85.80	0.44	YES
18*	Female	70	6	UCTD	30	MMF	91.84	0.41	NO
19	Female	35	132	SLE	50	HCQ,MMF	137.35	0.67	YES
20	Female	32	84	SLE	30	HCQ,MMF,LEF	29.61	0.09	YES
21*	Female	58	2	$MDA5^+$DM	50	CTX,HCQ,AZA	102.00	0.34	YES
22*	Male	40	3	$MDA5^+$DM	100	None	340.99	0.61	NO
23*	Female	65	3	pSS	50	CTX	103.23	0.37	YES
24*	Male	70	1.5	**anti-Jo-1 ab+DM**	100	HCQ	228.81	0.87	NO
25*	Female	39	3	SLE	50	CTX,Tac	99.12	0.42	YES
26*	Female	41	72	SLE	500	CsA	46.41	0.39	YES

27	Male	47	2	MDA5$^+$DM	60	CsA	165.30	0.57	YES
28	Female	65	48	RA	15	MTX,LEF	362.23	0.89	NO
29	Female	52	60	UCTD	60	HCQ,Tac	158.40	0.40	NO
30	Female	68	5	MDA5$^+$DM	60	CTX,HCQ,MMF	1.96	0.02	YES

*Probable case, others are etiology confirmed case
MDA5$^+$DM: Anti-MDA5-ab positive DM;
DM: dermatomyositis;
PM: polymyositis;
AAV:ANCA associated vasculitis;
SLE: Systemic lupus erythematosus;
AOSD:Adult onset Still's disease;
pSS:Primary Sjogren's syndrome;
UCTD:Undifferentiated connective tissue disease
HCQ:hydroxychloroquine;
CsA:Cyclosporine ;
Thd:Thalidomide;
Tac:Tacrolimus;
MMF:Mycophenolate Mofetil;
RTX:Rituximab;
AZA:Azathioprine;
MTX:Methotrexate;
LEF:Leflunomide;
